# SLC2A9 Is a High-Capacity Urate Transporter in Humans

**DOI:** 10.1371/journal.pmed.0050197

**Published:** 2008-10-07

**Authors:** Mark J Caulfield, Patricia B Munroe, Deb O'Neill, Kate Witkowska, Fadi J Charchar, Manuel Doblado, Sarah Evans, Susana Eyheramendy, Abiodun Onipinla, Philip Howard, Sue Shaw-Hawkins, Richard J Dobson, Chris Wallace, Stephen J Newhouse, Morris Brown, John M Connell, Anna Dominiczak, Martin Farrall, G. Mark Lathrop, Nilesh J Samani, Meena Kumari, Michael Marmot, Eric Brunner, John Chambers, Paul Elliott, Jaspal Kooner, Maris Laan, Elin Org, Gudrun Veldre, Margus Viigimaa, Francesco P Cappuccio, Chen Ji, Roberto Iacone, Pasquale Strazzullo, Kelle H Moley, Chris Cheeseman

**Affiliations:** 1 Clinical Pharmacology and The Genome Centre, William Harvey Research Institute, Barts and The London School of Medicine and Dentistry, London, United Kingdom; 2 Department of Physiology, University of Alberta, Edmonton, Alberta, Canada; 3 Department of Obstetrics and Gynaecology, Washington University in St Louis School of Medicine, St. Louis, Missouri, United States of America; 4 Department of Statistics, Pontificia Universidad Católica de Chile, Santiago, Chile; 5 Clinical Pharmacology Unit, University of Cambridge, Addenbrookes Hospital, Cambridge, United Kingdom; 6 Glasgow Cardiovascular Research Centre, University of Glasgow, Glasgow, United Kingdom; 7 Cardiovascular Medicine, University of Oxford, Wellcome Trust Centre for Human Genetics, Oxford, United Kingdom; 8 Centre National de Génotypage, Evry, France; 9 Cardiovascular Sciences, University of Leicester, Glenfield Hospital, Leicester, United Kingdom; 10 Epidemiology and Public Health, University College, London, United Kingdom; 11 National Heart Lung Institute and Epidemiology and Public Health, Imperial College, London, United Kingdom; 12 Institute of Molecular and Cell Biology, University of Tartu, Tartu, Estonia; 13 Department of Cardiology, University of Tartu, Tartu, Estonia; 14 Centre of Cardiology, North Estonia Medical Centre, Tallinn, Estonia; 15 Cardiovascular Medicine and Epidemiology, Clinical Sciences Research Institute, University of Warwick Medical School, Clifford Bridge Road, Coventry, United Kingdom; 16 Department of Clinical and Experimental Medicine, Federico II University of Naples Medical School, Naples, Italy; Peninsula Medical School, United Kingdom

## Abstract

**Background:**

Serum uric acid levels in humans are influenced by diet, cellular breakdown, and renal elimination, and correlate with blood pressure, metabolic syndrome, diabetes, gout, and cardiovascular disease. Recent genome-wide association scans have found common genetic variants of *SLC2A9* to be associated with increased serum urate level and gout. The *SLC2A9* gene encodes a facilitative glucose transporter, and it has two splice variants that are highly expressed in the proximal nephron, a key site for urate handling in the kidney. We investigated whether SLC2A9 is a functional urate transporter that contributes to the longstanding association between urate and blood pressure in man.

**Methods and Findings:**

We expressed both *SLC2A9* splice variants in Xenopus laevis oocytes and found both isoforms mediate rapid urate fluxes at concentration ranges similar to physiological serum levels (200–500 μM). Because SLC2A9 is a known facilitative glucose transporter, we also tested whether glucose or fructose influenced urate transport. We found that urate is transported by SLC2A9 at rates 45- to 60-fold faster than glucose, and demonstrated that SLC2A9-mediated urate transport is facilitated by glucose and, to a lesser extent, fructose. In addition, transport is inhibited by the uricosuric benzbromarone in a dose-dependent manner (*K*_i_ = 27 μM). Furthermore, we found urate uptake was at least 2-fold greater in human embryonic kidney (HEK) cells overexpressing SLC2A9 splice variants than nontransfected kidney cells. To confirm that our findings were due to SLC2A9, and not another urate transporter, we showed that urate transport was diminished by *SLC2A9*-targeted siRNA in a second mammalian cell line. In a cohort of men we showed that genetic variants of *SLC2A9* are associated with reduced urinary urate clearance, which fits with common variation at *SLC2A9* leading to increased serum urate. We found no evidence of association with hypertension (odds ratio 0.98, 95% confidence interval [CI] 0.9 to 1.05, *p* > 0.33) by meta-analysis of an *SLC2A9* variant in six case–control studies including 11,897 participants. In a separate meta-analysis of four population studies including 11,629 participants we found no association of *SLC2A9* with systolic (effect size −0.12 mm Hg, 95% CI −0.68 to 0.43, *p* = 0.664) or diastolic blood pressure (effect size −0.03 mm Hg, 95% CI −0.39 to 0.31, *p* = 0.82).

**Conclusions:**

This study provides evidence that SLC2A9 splice variants act as high-capacity urate transporters and is one of the first functional characterisations of findings from genome-wide association scans. We did not find an association of the *SLC2A9* gene with blood pressure in this study. Our findings suggest potential pathogenic mechanisms that could offer a new drug target for gout.

## Editors' Summary

**Background.:**

Blood is continually pumped around the human body to deliver the chemicals needed to keep the body's cells alive and to take cellular waste products to the kidneys where they are filtered out of the blood and excreted in the urine. In healthy people, the levels of nutrients and waste products in serum (the liquid part of blood) fall within “normal” ranges but in ill people these levels can be very different. For example, serum uric acid (urate) levels are usually increased in people with gout. In this arthritic condition, uric acid crystallizes in the joints (often those in the big toe) and causes swelling and intense pain. Increased serum urate levels, which are also associated with high blood pressure, diabetes, and several other important conditions, can be caused by eating food that is rich in chemicals called purines (for example, liver, dried beans, and port). The body also converts its own purines into uric acid so genetic variations in the enzymes involved in purine breakdown can alter serum urate levels, as can variations in the rate of urate removal from the body by the kidneys. Urinary urate excretion is controlled by urate transporters, proteins that carry urate into and out of the kidney cells. Uricosuric drugs, which are used to treat gout, reduce serum urate levels by inhibiting a urate transporter that reabsorbs urate from urine.

**Why Was This Study Done?:**

Several urate transporters have already been identified but recently, using an approach called genome-wide association scanning, scientists found that some genetic variants of a human gene called *SLC2A9* are more common in people with high serum urate levels than in people with normal levels. *SLC2A9* encodes a glucose transporter (a protein that helps to move the sugar glucose through cell membranes) and is highly expressed in the kidney's main urate handling site. Given these facts, could SLC2A9 (the protein made from *SLC2A9*) be a urate transporter as well as a glucose transporter? In this study, the researchers investigate this possibility and also ask whether genetic variations in *SLC2A9* might be responsible for the association between serum urate levels and high blood pressure.

**What Did the Researchers Do and Find?:**

The researchers first expressed *SLC2A9* in frog eggs, a type of cell that does not have its own urate transporter. They found that urate rapidly moved into eggs expressing *SLC2A9* but not into control eggs, that SLC2A9 transported urate about 50 times faster than glucose, and that glucose stimulated SLC2A9-mediated urate transport. Similarly, overexpression of *SLC2A9* in human embryonic kidney cells more than doubled their urate uptake. Conversely, when the researchers used a technique called RNA interference to reduce the expression of mouse SLC2A9 in mouse cells that normally makes this protein, urate transport was reduced. Next, the researchers looked at two small parts of *SLC2A9* that vary between individuals (so-called single polynucleotide polymorphisms) in nearly 900 men who had had their serum urate levels and urinary urate excretion rates measured. They found that certain genetic variations at these two sites were associated with increased serum urate levels and decreased urinary urate excretion. Finally, the researchers used a statistical technique called meta-analysis to look for an association between one of the *SLC2A9* gene variants and blood pressure. In two separate meta-analyses that together involved more than 20, 000 participants in several studies, there was no association between this gene variant and blood pressure.

**What Do These Findings Mean?:**

Overall, these findings indicate that SLCA9 is a high capacity urate transporter and suggest that this protein plays an important part in controlling serum urate levels. They provide confirmation that common genetic variants in *SLC2A9* affect serum urate levels to a marked degree, although they do not show exactly which genetic variant is responsible for increasing serum urate levels. They also provide important new insights into how the kidneys normally handle urate and suggest ways in which this essential process may sometimes go wrong. Thus, these findings could eventually lead to new treatments for gout and possibly for other diseases that are associated with increased serum urate levels.

**Additional Information.:**

Please access these Web sites via the online version of this summary at http://dx.doi.org/10.1371/journal.pmed.0050197.

- The UK National Health Service Direct health encyclopedia provides detailed information for patients about gout
- MedlinePlus provides links to many sources of information about gout (in English and Spanish), including “What is gout?”, an easy-to-read guide from the US National Institutes of Arthritis and Musculoskeletal and Skin Diseases
- Wikipedia also has pages on gout, uric acid, and SCL2A9 (note: Wikipedia is a free online encyclopedia that anyone can edit; available in several languages)
- The Arthritis Research Campaign also has information on gout

## Abstract 3

Mark Caulfield and colleagues show that the*SLC2A9* gene, which encodes a facilitative glucose transporter, is also a high-capacity urate transporter.

## Introduction

Elevated serum urate levels are associated with important common disorders such as gout, metabolic syndrome, diabetes, hypertension, and cardiovascular morbidity and mortality [1–4]. Uric acid is principally derived from the breakdown of dietary and cellular purines. Humans and great apes are exposed to higher urate levels than other mammalian species because of the inactivation of hepatic uricase [5]. In humans the kidney has a pivotal role in urate handling, with secretory mechanisms balanced against efficient reabsorption resulting in only 10% of the filtered load actually being excreted in the urine [5]. The established urate transporter systems in the proximal nephron includes; the urate anion transporter (URAT1), which is a target of uricosuric drugs, multiple organic anion transporters (OATs 1–4), the urate transporter (UAT), and a voltage dependent organic anion transporter (OATv1) [5].

It is possible that genetic variation in either enzymatic breakdown of purines or renal transporters of uric acid might elevate serum levels and account for the long-standing association with blood pressure and common cardiovascular phenotypes [1–5]. Recently, two separate genome-wide association scans identified and replicated association of serum urate level with common variants within the glucose transporter *SLC2A9* (*GLUT9*) gene region on Chromosome 4 [6,7]. Interestingly, this member of the facilitative glucose transporter family has two splice variants most strongly expressed in the apical and basolateral membranes of the proximal tubular epithelial cells of the kidney [8,9]. SLC2A9 is not as efficient a glucose transporter as GLUT1 and GLUT4 [8], so we set out to test whether SLC2A9a and SLC2A9b splice variants act as urate transporters. We used data from the Olivetti Heart Study to test association of *SLC2A9* variants and fractional urate excretion, and then tested for association with blood pressure and hypertension in additional cohorts.

## Methods

### Reagents and Antibodies

All chemicals were obtained from Sigma-Aldrich unless otherwise noted. Two polyclonal antibodies against N-terminal peptides of both splice variants of human SLC2A9 were raised in rabbits (Rockland Immunochemicals). The SLC2A9a antibody was raised against DTSHARPPGPGRALLEC and the SLC2A9b was raised against KSRGEDEESDSAKKC. Both antibodies were peptide purified before use. Two polyclonal antibodies against N-terminal peptides of both splice variants of mouse SLC2A9 were also raised in rabbits (Rockland Immunochemicals). The mSLC2A9a antibody was raised against MDSRELALASLMC and the mSLC2A9b antibody was raised against MKLSEKNSAETKESC, and both antibodies were peptide purified before use.

### mRNA Preparation and Xenopus laevis Oocyte Microinjection

Plasmids containing genes encoding the wild-type human SLC2A9a or SLC2A9b isoform were linearized with Nhe I and transcribed in vitro with T7 polymerase mMESSAGE mMACHINE (Ambion). Adult female X. laevis oocytes (prepared as described in [9]) were injected with 20 nl (1 ng/nl) SLC2A9a or SLC2A9b synthetic mRNA transcript and incubated for 5 d at 16–18 °C prior to functional uptake assays. The concentration of RNA prior to injection was determined using Bio-Rad SmartSpec 3000 machine.

### Determination of Functional Activity by Radiotracer Flux Assays

The influx experiments were performed at 22 °C using 10–12 oocytes for each condition and ^14^C-labelled urate at a specific activity of 54 mCi/mmole, 250 μCi/ml. Oocytes were washed with ice-cold modified Barth's medium (MBM) to stop the uptake, and then individual oocytes were placed in vials and dissolved in 0.5 ml of 5% SDS for 30 min. Finally, scintillation fluid (5 ml) was added to each vial and radioactivity measured using a Beckman LS6500 liquid scintillation counter. All experiments were performed three to six times and the results were corrected for the flux values obtained with non-injected oocytes obtained from the same frog.

Efflux experiments were performed by injecting oocytes with 40 nl of ^14^C-urate just prior to the flux measurements. Eggs were then incubated in batches of 20 in 1 ml of medium for 20 min at 22 °C. Samples of 20 μl of incubation medium were taken every 2 min to measure the appearance of urate in the outside solution. The incubation volume was kept constant by sequential addition of medium after the removal of each sample. The activity remaining in the eggs at the end of the incubation was measured by solubilising the oocytes in 1 ml of 5% SDS overnight. Incubation medium consisted of MBM supplemented with 5 mM D-glucose, D-fructose, uric acid, or L-glucose (control). Efflux data were then plotted as a function of the fraction of total urate remaining in the eggs at each incubation time point. This was expressed as the log (remaining urate/initial injected urate × 100) for the SLC2A9a-expressing eggs. Net SLC2A9a-mediated efflux was determined by subtracting urate efflux observed in non-injected oocytes from total urate efflux from SLC2A9a oocytes at each time point.

### Thin-Layer Chromatography

To determine if urate was metabolized within the *Xenopus* oocytes we microinjected 40 nl of ^14^C- labelled 2 mM urate and collected the efflux medium from both water-injected eggs and those expressing SLC2A9a. We then loaded 100 μl of the efflux media onto a silica-coated glass chromatography plate and eluted with a 80:15:5 n-propanol:ammonium hydroxide (25%):water solution. Standard urate and allantoin solutions were used for comparison. After drying the plate, a vanillin spray was used to visualize the urate and allantoin. The *R*_f_ value (distance travelled from solvent front) for urate was 0.0103 and that for allantoin 0.238.

### Kinetic Analysis

Urate transport into oocytes expressing SLC2A9a or SLC2A9b was measured over a range of concentrations from 0.001 to 2 mM using 20 min incubations, which had been determined to be within the linear component of uptake. Uptake was corrected for nonspecific entry using non-mRNA injected eggs from the same batch in each experiment. SIGMAPLOT 6 software was used to determine the transport kinetics for the SLC2A9 mediated urate uptake by nonlinear regression analysis. The analysis assumed standard simple Michaelis-Menten kinetics for a facilitative transporter with no cooperativity. In addition to the regression analysis, an Eadie-Hofstee linear transformation was included, the results of which could imply that a more complex kinetic model is required to fully describe the characteristics of this transporter. Inhibitors were used at concentrations similar to previous studies of other glucose or urate transporters, e.g., phloretin, benzbromarone, and furosemide, and which corresponded with effective pharmacological doses, e.g., probenecid [10].

### HEK293 Cells and Urate Uptake

HEK293 cells were stably transfected with human SLC2A9a or SLC2A9b as shown previously [8]. These cells were grown to 90% confluency in T75 flasks. The cells were then washed with EDTA, trypsinised, decanted into 15 ml conical tubes, and centrifuged for 4 min at 1,100 rpm at 4 °C. The supernatant was removed and the cells resuspended in 4 ml of Dulbecco's modified Eagle medium (DMEM) with 2% serum. After incubation for 2 h at 37 °C while shaking at 120 rpm, the cells were centrifuged for 4 min at 1,100 rpm at 4 °C and the supernatant removed. Next, the cells were washed twice in Kreb's Ringer phosphate (KRP) buffer, centrifuged for 4 min at 1,100 rpm at 4 °C and resuspended in 0.450 ml of KRP buffer with 50 μl of 1.2 mM ^14^C-labeled urate (Moravek Biochemicals). The cells were then incubated at 37 °C with shaking at 120 RPM for 6 min, after which they were placed on ice for 5 min. Finally, the supernatant was removed after centrifugation and the pellet washed twice with cold KRP buffer, before lysis. After incubation in lysis media for 30 min on ice, a final centrifugation step at 1,100 rpm for 1 min was performed, a 500 μl aliquot of the supernatant was added to scintillation fluid (5 ml) and the radioactivity measured using a Beckman LS6500 liquid scintillation counter. The remainder of the supernatant was used for protein quantitation and Western immunoblot. All experiments were done at least three times.

### Western Immunoblot Analysis

Cell lysates were solubilised in Laemmli buffer and subjected to SDS-PAGE, transferred onto nitrocellulose membranes, blocked with 5% dry milk in Tris-buffered saline/Tween 20 (TBS-T), and probed with an antibody raised against the human SLC2A9a N terminus (1.5 μg/ml in 1% dry milk/TBS-T), or the human SLC2A9b N terminus (1.0 μg/ml in 1% dry milk/TBS-T). For the murine insulinoma cell (MIN6) lysates, polyclonal antibodies to the mouse SLC2A9a and SLC2A9b, previously described, were used [11]. Blots were then probed with a horseradish peroxidase-coupled goat anti-rabbit secondary antibody (Santa Cruz Biotechnology) and detection was performed using the SuperSignal Dura Western kit (Pierce Biotechnology). Blot quantitation after scanning was carried out using NIH Image.

### Culture of Mouse Insulinoma Cells (MIN6)

MIN6 [12] cells were maintained in DMEM containing 25 mM glucose, supplemented with 15% heat-inactivated foetal bovine serum, 50 μM β-mercaptoethanol, 100 U/ml penicillin,100 μg/ml streptomycin, 100 μg/ml L-glutamine, 10 mM HEPES, and 1 mM sodium pyruvate, in 5% CO_2_, 95% air at 37 °C. MIN6 cells were used between passages 20 and 30. The radiolabelled urate uptake assays were conducted as described above for the HEK293 cells. For the cold urate competition assays, 50 μl of cold uric acid stock at a concentration of 1 mM dissolved in Tris buffer (pH 8.1) was added to the KRP buffer with labelled urate to give a final concentration of 1 mM unlabeled urate. The remainder of the assay was performed as described above.

### SLC2A9 siRNA Transfection

The polyamine transfection reagent, Trans IT- TKO (Mirus) was used to transfect the insulinoma cells according to the manufacturer's instructions. The mouse *SLC2A9* siRNA and scrambled siRNA (negative control) were obtained from Ambion. Cells were transfected for 72 h for protein isolation. Radiolabelled urate uptake assays were conducted as described above.

### Association Studies with Serum Urate, Fractional Urate Excretion, Blood Pressure, and Hypertension: Participants

#### The Olivetti Heart Study.

The Olivetti Heart Study population is derived from the male work force of the Olivetti factories of Pozzuoli (Naples) and Marcianise (Caserta), Italy. The general characteristics of the study and its methodological procedures have been described [12,13]. The local ethics committee approved the study protocol, and informed consent was obtained from all participants. A total of 1,085 individuals aged 25–74 y were examined in 1994–1995: of these, 907 (83.6%) were seen again in 2002–2004, and of these, 868 individuals had DNA available for genotyping at both time points [14]. We analysed individuals at both time points for association with serum urate and fractional excretion of uric acid and the 1994–1995 dataset only for association with blood pressure and hypertension.

#### Whitehall II study.

After ethical clearance the Whitehall study enrolled 10,308 subjects (3,413 women) aged 35–55 y working in the London offices of 20 civil service departments between 1985–1988. In this longitudinal study, blood pressure was recorded at phase 1 (1985–1988), phase 3 (1991–1993), phase 5 (1997–1999), and phase 7 (2003–2004). DNA was stored from phase 7 of the study. For association testing with blood pressure and hypertension we selected individuals from phase 5 as diabetes ascertainment and blood pressure medication records were most complete from this phase. For case–control analyses, hypertensive participants were selected using the following criteria: blood pressure recordings of ≥ 145/95 mm Hg, prescribed antihypertensive medication, or a physician diagnosis of hypertension. Normotensive control participants were selected on the basis of blood pressure recordings ≤ 130/85 mmHg and not taking any antihypertensive medications.

#### English Longitudinal Study of Ageing (ELSA).

After ethical approval the participants were drawn from around 12,000 respondents to the Health Survey for England (HSE) over three separate years (1998, 1999, and 2001) to provide a representative sample of the English population aged 50 y and over. Each individual had a mean of three blood pressure measures taken when the participant was seated, and antihypertensive medications were recorded, DNA was extracted from 5,672 participants in wave 2 (2004). For association testing with blood pressure and hypertension we selected individuals from wave 2. Cases and normotensive controls were defined using the same criteria as the Whitehall II study.

#### BRIGHT study.

The MRC BRIGHT study (http://www.brightstudy.ac.uk/) comprises 2,500 hypertensive participants and 2,000 normotensive controls of white European ancestry. Case ascertainment and phenotyping has been described previously [15]. Briefly, cases were included if they had blood pressure readings ≥ 150/100 mm Hg based on one reading or ≥ 145/95 mm Hg based on the mean of three readings. Healthy age- and sex-matched normotensive controls (< 140/90 mm Hg) were recruited using the same strict selection criteria. Ethics Committee approval was received from the multi- and local-research committees, and all participants gave informed written consent.

#### London Life Sciences Prospective Cohort Study.

The London Life Sciences Prospective Cohort Study (LOLIPOP) is a prospective study of potentially up to 24,000 participants (UK-based individuals of Indian and white European ancestry) recruited primarily for investigating cardiovascular risk factors. For this study we selected white European individuals—485 hypertensive cases and 458 normotensive controls—drawn from the top and bottom 10% of the blood pressure distribution. All blood pressure readings were off-medication.

#### The Estonian HYPEST sample collection.

The Estonian participants were recruited during 2004–2007 across the entire country in the framework of the HYPEST sample collection (*n* = 1,823) targeting hypertension risk factors in the Estonian population (permissions no 122/13, 22.12.2003; 137/20, 25.04.2005 by Ethics Committee on Human Research of University of Tartu, Estonia). Hypertensive patients were recruited at the North Estonia Medical Center, Tartu Estonia. Healthy (exclusion criteria: cardiovascular disease, diabetes, and antihypertensive treatment), normotensive individuals were recruited across the whole country. The majority of the HYPEST participants (*n* = 1,482) possess a documented history of multiple systolic blood pressure (SBP) and diastolic blood pressure (DBP) readings. For this study we defined cases (*n* = 596) as individuals with either blood pressure readings ≥ 160/100 mm Hg based on the median of several measurements or under antihypertensive therapy. Controls (*n* = 650) were defined as having median blood pressure readings below 140/90 mm Hg. The quantitative association analysis of SBP and DBP (*n* = 1,284) included both untreated (*n* = 881) and treated individuals (*n* = 403).

### Genotyping

Two variants of *SLC2A9* (SNPs rs7442295 and rs13113918) were investigated in the Olivetti Study for association with serum urate and fractional urinary urate excretion. For association analyses with blood pressure and hypertension, one intragenic SNP (rs13113918) was genotyped in each cohort. SNP genotyping in Olivetti, Whitehall II, and ELSA participants was performed with TaqMan assay (Applied Biosystems) followed by allelic discrimination using the ABI PRISM 7900HT Sequence Detection System and software (SDSv2.0, Applied Biosystems) [16]. SNP genotyping in BRIGHT, HYPEST, and LOLIPOP subjects was performed using the KASPar chemistry, a competitive allele-specific PCR SNP genotyping system using FRET quencher cassette oligonucleotides [17]. SNPs were checked for departure from Hardy-Weinberg equilibrium, low minor allele frequency (<1%), and high level of missing data. Allele frequencies at both sites were similar to those previously reported in Hapmap [18].

### Statistical Analysis

For the Olivetti study, between-group comparisons were performed using unpaired *t*-tests for serum urate and fractional uric acid excretion. Analysis of covariance was used to model age and body mass index (BMI) covariation.

In order to test for associations with SNP rs13113918 we performed linear regression for the quantitative phenotypes (SBP and DBP) in Olivetti, Whitehall II, ELSA, and HYPEST reported as effect size in mm Hg with 95% confidence intervals. We used logistic regression for the qualitative phenotype hypertension (HYP) in all the studies reported as an odds ratio with 95% confidence intervals. Age, gender, and BMI were included as covariates in all models. We tested additive models of inheritance using either PLINK software [19] or SAS v9.1. For individuals on antihypertensive medication, we adjusted systolic and diastolic blood pressure measures by adding 15 mm Hg to systolic and 10 mm Hg to diastolic readings [20]. Individuals with BMI > 35 and those with diabetes were excluded from all analyses.

Inverse-variance weighted meta-analysis was performed using the “metan” [21] procedure in Stata 10.0.

## Results

### Human SLC2A9 Is a High-Capacity Urate Transporter

**Figure 1:**
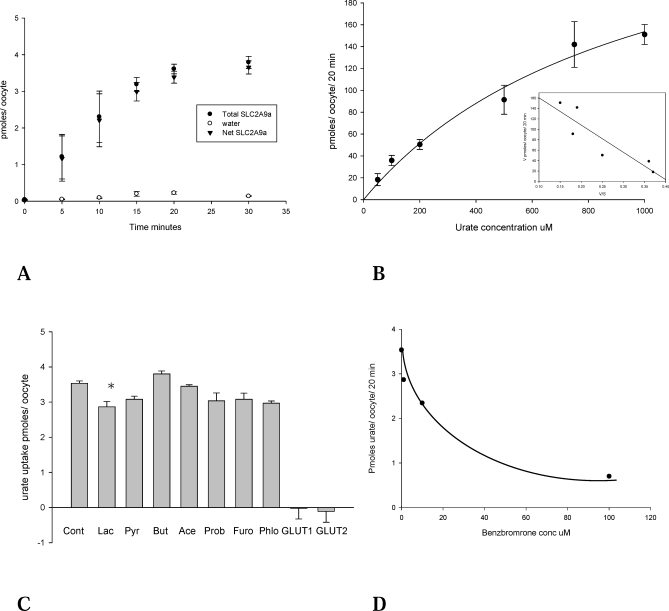
Characterisation of Urate Fluxes and Kinetics Mediated by Human SLC2A9 Expressed in Xenopus laevis Oocytes and the Effect of Short-Chain Fatty Acids and Uricosurics on Urate Transport (A) Time course of SLC2A9a-mediated urate uptake into *Xenopus* oocytes. Oocytes were injected with *SLC2A9a* cRNA or water and 4 d later incubated with 10 μM urate at 22 °C for the time periods indicated. Symbols represent the average uptake into ten oocytes per time point, with error bars representing the standard error of the mean (SEM). Solid circles show total uptake into oocytes expressing *SLC2A9a* cRNA, open circles urate uptake into water-injected oocytes, and inverted triangles the net uptake obtained by subtracting the water data from the total uptake for each time point. (B) Kinetics of human SLC2A9-mediated urate uptake in *Xenopus* oocytes. Symbols represent the mean data from six separate experiments each using ten oocytes per substrate concentration. Uptake was measured in oocytes injected with either *SLC2A9a* or *SLC2A9b* cRNA 4 d prior and corrected for uptake into water-injected oocytes. The curve was fitted by nonlinear regression analysis. The *K*_m_ = 981 μM and the *V*_max_ = 304 pmol/oocyte/20 min. Insert shows an Eadie-Hofstee plot of the same data. (C) Effect of uricosemics and short chain fatty acids on human SLC2A9a mediated urate uptake. *Xenopus* oocytes were injected with water or *SLC2A9a* cRNA and urate uptake measured 4 d later. Oocytes were incubated with 10 μM urate for 20 min, and bars represent net uptake determined by subtracting uptake into water-injected eggs from total uptake into *SLC2A9a* expressing oocytes, error bars are the SEM. Note: at no time was the urate uptake into the water-injected eggs more than 11% of the total uptake into *SLC2A9*-expressing oocytes. Compounds used were: no additional reagent (Cont), lactate 1 mM (Lac), pyruvate 1 mM (Pyr), butyrate 1 mM (But), acetate 1 mM (Ace), probenecid 1 mM (Prob), furosemide 100 μM (Furo), and phloretin 1 mM (Phlo). For comparison, a separate series of oocytes were injected with *SLC2A1* (GLUT1) or *SLC2A2* (GLUT2) cRNA and 100 μM urate uptake measured 4 d later using 30 min incubation times. Bars represent net uptake corrected for uptake into water-injected oocytes. *Significant inhibition at *p* ≤ 0.05. (D) Dose-dependent effect of benzbromarone on SLC2A9a-mediated urate uptake. Oocytes were injected with *SLC2A9a* cRNA or water and 4 d later were incubated with 10 μM urate for 20 min in the presence of 0, 1, 10, or 100 μM benzbromarone. Points represent the mean net uptake into ten oocytes corrected for uptake into water-injected eggs. Error bars were smaller than the data points.

To test whether *SLC2A9* splice variants act as urate transporters we separately microinjected synthetic human SLC2A9a and SLC2A9b mRNA transcripts into X. laevis oocytes and measured uptake or efflux of radiolabelled urate**.** Both proteins were readily expressed in the oocyte plasma membrane within a couple of days as confirmed by immunohistochemistry (Figure S1). However, we studied the oocytes 4 d after mRNA injection so that urate fluxes could be determined under the same conditions as previously published for SLC2A9 mediated hexose transport. We found that human SLC2A9a and SLC2A9b mediated very rapid urate fluxes which necessitated incubation for only 20 min in subsequent kinetic and inhibition experiments (Figure 1A). In contrast, uptake of urate into non-injected eggs was very slow, indicating negligible endogenous transport activity for this substrate. Transport was then measured over a range of urate concentrations, which bracketed the normal human physiological plasma concentrations (200–500 μM). Both human SLC2A9a- and SLC2A9b-mediated urate fluxes showed saturation and were identical, so data for the two splice variants were combined for kinetic analysis. Figure 1B shows the averaged data from six such experiments and nonlinear regression analysis was used to fit a Michaelis-Menten function with a *K*_m_ of 981 μM and a *V*_max_ of 304 pmol/oocyte/20 min. The insert shows the Eadie-Hofstee plot for the same data. In contrast, we were unable to detect any urate flux (100 μM) mediated by either human GLUT1 (SLC2A1) or GLUT2 (SLC2A2), both class I facilitative glucose transporters (Figure 1C).

### Transport of Urate by SLC2A9 Shows Limited Sensitivity to Uricosurics

A number of compounds known to promote urate loss in the urine via inhibition of other renal urate transporters such as URAT1 were tested to determine if they reduced SLC2A9 mediated urate transport. Benzbromarone showed a dose-responsive inhibition with 10 μM reducing urate uptake by 34% and 100 μM by 80% (Figure 1D). Additional kinetic analyses using two concentrations of urate (10 and 100 μM) and a Dixon analysis showed that the *K*_i_ for benzbromarone inhibition of urate transport mediated by SLC2A9a was 27 μM. However, probenecid at a concentration of 1 mM had no significant effect on urate uptake, *p* > 0.05 (Figure 1C). Similarly, furosemide had no significant effect at 100 μM, and, of the short chain fatty acids lactate, pyruvate, butyrate, and acetate at a concentration of 1 mM, only lactate induced a small and just significant inhibition, *p* < 0.05 (Figure 1C). Finally, the class I hexose transporter inhibitor phloretin had no significant effect on urate transport (< 10% inhibition) when applied at a concentration of 1 mM, *p* > 0.05 (Figure 1C).

### SLC2A9 Mediates Exchange of Urate for Glucose or Fructose

**Figure 2:**
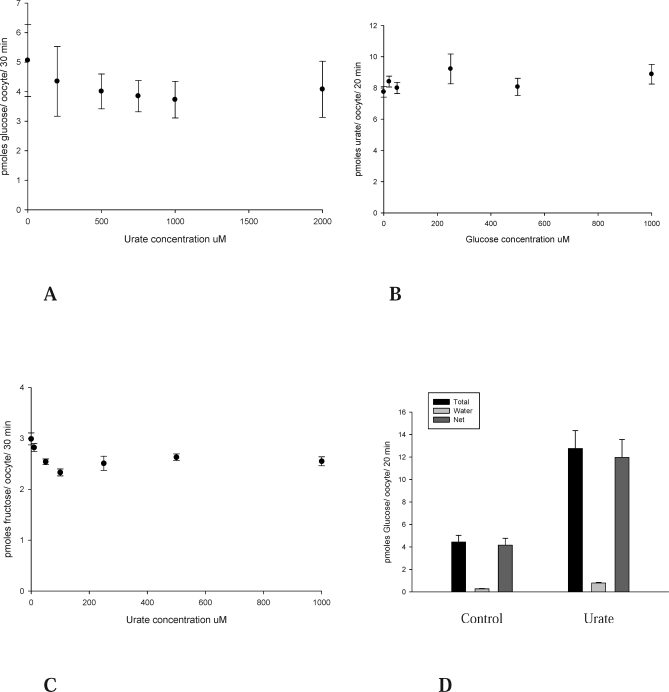
Interaction between Hexoses and Human SLC2A9a-Mediated Urate Uptake in *Xenopus* Oocytes Oocytes were injected with *SLC2A9a* cRNA 4 d prior to uptake experiments. Symbols represent mean net substrate uptake and error bars the SEM for measurements made in 6–10 oocytes per condition. Total uptake of substrates was measured into *SLC2A9a* cRNA-injected oocytes and then corrected for the uptake measured under identical conditions using water injected oocytes from the same batch of eggs. (A) Uptake over 30 min of 50 μM glucose in the presence of increasing concentrations of urate. (B) Uptake over 20 min of 5 μM urate in the presence of increasing concentrations of glucose. (C) Uptake over 30 min of 50 μM fructose in the presence of increasing concentrations of urate. (D) Human SLC2A9a-mediated glucose and urate exchange. Oocytes injected with *SLC2A9a* cRNA or water, 4 d prior, were incubated with non-radiolabelled 2 mM L-glucose or 2 mM urate for 1 h. Oocytes were then washed and then incubated at 22 °C for 30 min in ^14^C-labeled 10 μM D-glucose. Bars represent the average total uptake into 20 eggs expressing *SLC2A9a* or water-injected eggs, and the difference between the two, the net uptake. Error bars represent the SEM.

**Figure 3:**
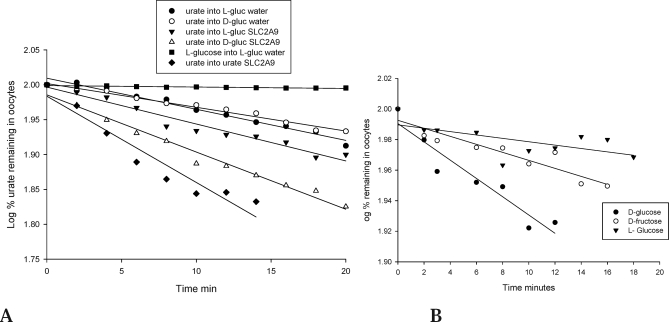
Human SLC2A9a-Mediated Urate Efflux from *Xenopus* Oocytes (A) Comparison of urate and L-glucose efflux from SLC2A9a-expressing oocytes. Oocytes injected with *SLC2A9a* cRNA 4 d prior (triangles) or water-injected eggs (circles or squares) were injected with ^14^C-labelled urate (circles, triangles, or diamonds) or ^14^C-labelled L-glucose (filled squares) to give an estimated initial intracellular concentration of 200 μM. 20 oocytes per condition were incubated at 22 °C in efflux medium, which was sampled every 2 min. Efflux media contained 5 mM D-glucose (open circles), 5 mM L-glucose (filled squares, circles or triangles), or 2 mM urate (filled diamonds). Data points represent the log percentage of urate or L-glucose remaining in the oocytes for each time point. Lines were fitted by linear regression. (B) Acceleration of SLC2A9a mediated urate efflux by extracellular D-glucose or D-fructose. Oocytes injected with *SLC2A9a* cRNA 4 d prior (triangles) or water-injected eggs were injected with ^14^C-labelled urate to give an estimated intracellular concentration of 200 μM. 20 oocytes per condition were incubated at 22 °C in efflux medium, which was sampled every 2 min. Efflux media contained 5 mM D-glucose (filled circles), 5 mM L-glucose (filled triangles) or 5 mM D-fructose (open circles). Data points represent the log percentage of urate remaining in the oocytes for each time point corrected for the efflux of urate from the water-injected eggs under the same conditions. Lines were fitted by linear regression.

Because SLC2A9a and SLC2A9b have previously been characterized as high-affinity, low-capacity glucose and fructose transporters, we tested the ability of these two hexoses to inhibit urate fluxes and, conversely, for urate to inhibit hexose uptake. Surprisingly, concentrations of glucose up to 1 mM had no effect on the transport of 10 μM urate (Figure 2B), and urate concentrations of up to 2 mM had no effect on the uptake of 50 μM D-glucose or D-fructose (Figure 2A and 2C). Note that the rates of urate transport were significantly greater than those for glucose. Urate fluxes were measured at 5 or 10 μM for only 20 min, whereas glucose rates were determined using 50 μM substrate for 30 min. Thus, converting flux rates to the same time period and equivalent concentrations suggest that urate is transported by SLC2A9 at rates 45- to 60-fold faster. The lack of significant competition between the hexoses and urate suggests that urate binds to a site on SLC2A9 that is different from the binding site for the hexoses. However, it could be argued that the injection of SLC2A9 mRNA was inducing expression of an endogenous protein that could separately mediate urate uptake. Therefore, we attempted to determine whether SLC2A9 could exchange hexoses with urate, providing further support for a single pathway mechanism. Since glucose and fructose are both rapidly phosphorylated upon entry into the oocyte, only urate efflux could be used to give a meaningful estimate of exchange rates. Oocytes were injected with radiolabelled urate and the efflux determined in the presence of 5 mM extracellular D-glucose, D-fructose, L-glucose, or 2 mM urate. Urate efflux could be described by a single exponential curve over a period of up to 20 min, and the presence of extracellular D-glucose greatly accelerated urate movement, while fructose did so to a lesser degree (7-fold versus 3-fold, respectively, Figures 3A and 3B). Extracellular cold urate accelerated the efflux of radiolabelled urate to the greatest degree, while extracellular L-glucose had no effect. Thin-layer chromatography was used to analyze the efflux medium, and only urate and no allantoin were detected on the plate for both water-injected eggs and those expressing SCL2A9a. Thus, SLC2A9 exhibits classical exchange *trans*-stimulation between urate and glucose or fructose. The rate of loss of injected radiolabelled L-glucose into the efflux medium was negligible, indicating that the injection protocol did not result in a nonspecific leakage of the substrate from the oocytes (Figure 3A).

The exchange of urate for glucose was confirmed by preloading the oocytes with 2 mM cold urate for 1 h and then, after washing, to remove extracellular urate, measuring the uptake of 100 μM D-glucose. The influx of D-glucose was increased 3-fold by intracellular urate in SLC2A9a-expressing eggs compared to preincubation with 2 mM L-glucose (Figure 2D). These data confirm that SLC2A9a can exchange extracellular glucose for intracellular urate.

### Increased Urate Uptake by Kidney Cells Overexpressing *SLC2A9* Isoforms

**Figure 4:**
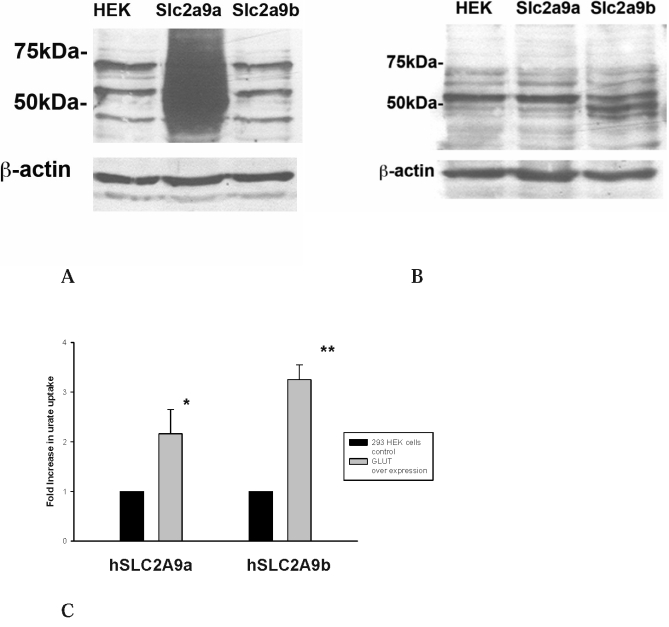
Specificity of Human SLC2A9a and SLC2A9b Antibodies and Urate Uptake into SLC2A9 Transfected Human Embryonic Kidney Cells (A) Western blotting of hSLC2A9 expressed in HEK cells. Expression of *hSLC2A9a* was detected as a broad band at approximately 50 kDa by a polyclonal antibody raised against the N terminus of *SLC2A9a* in HEK293 cells overexpressing *SLC2A9a* (middle lane, Slc2a9a) but not detected in cells overexpressing *SLC2A9b* (right lane, Slc2a9b), or nontransfected HEK cells (left lane, HEK). (B) Expression of hSLC2A9b in transfected HEK293 cells. *hSLC2A9b* was detected by a polyclonal antibody raised against the N terminus of *hSLC2A9b* in HEK293 cells overexpressing *hSLC2A9b* (right lane, Slc2a9b), but not detected in cells overexpressing *hSLC2A9a* (middle lane, Slc2a9a). (C) Increase in urate uptake in HEK293 cells overexpressing either hSLC2A9a or hSLC2A9b. Radiolabelled urate uptake was measured in human embryonic kidney cells, which were stably overexpressing *SLC2A9a* or *SLC2A9b* as compared to their respective nontransfected controls. Uptakes of 120 μM urate were measured over 6 min at 37 °C. *p < 0.05; **p < 0.02.

Both overexpressed proteins appear as a smear of glycosylated bands between 37 and 50 kDa, as previously reported using a different polyclonal antibody to the C terminus that was unable to distinguish SLC2A9a from SLC2A9b [8,21]. Previous studies had also already demonstrated plasma membrane expression of human (h) SLC2A9 in these cell lines [8]. In order to check the specificity of each antibody, total protein from cells overexpressing the different splice variants were loaded side by side in polyacrylamide gels and immunoblotted with the two antibodies separately. Each individual antibody detected the correct overexpressed protein but not the alternative splice variants. Both SLC2A9a and SLC2A9b were expressed at low levels endogenously in HEK293 cells as seen by the multiple bands in lanes 1 and 3 of Figure 4A and lanes 1 and 2 of Figure 4B. In prior studies we have demonstrated that these multiple bands in HEK cells reduce to one band at 37 kDa upon deglycosylation [8]. In the presence of preimmune sera (unpublished data) no specific bands were detected.

We observed that radiolabelled uric acid was transported into the HEK293 cells stably overexpressing either human SLC2A9a or SLC2A9b at the cell surface. The rate of urate uptake was at least 2-fold greater than for control HEK293 cells (Figure 4C).

### Confirmation of SLC2A9-Mediated Urate Transport in a Second Mammalian Cell Line

**Figure 5:**
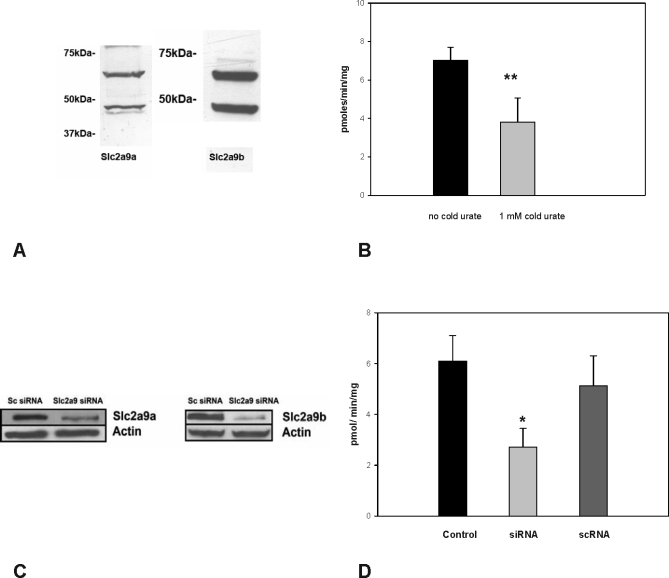
Expression of *SLC2A9* and Urate Uptake into Mouse Insulinoma MIN6 Cells (A) Western blotting of mouse *SLC2A9* in MIN6 cells. Cell lysates were analyzed by Western blot using antibodies to murine *SLC2A9a* and *SLC2A9b* of MIN6 cell lysates. (B) ^14^C-urate uptake into MIN6 cells is competitively inhibited by cold urate. Radiolabelled urate uptake was measured in mouse insulinoma cells, which endogenously express *mSLC2A9a* or m*SLC2A9b*. Uptake of ^14^C-urate, 120 μM, was measured for 6 min at 37 °C in the presence or absence of an additional 1mM cold urate. ** *p* < 0.02. (C) *SLC2A9a* and *SLC2A9b* expression is reduced by treatment of MIN6 cells with siRNA specific for *mSLC2A9*. Cells were treated with either scrambled RNA (scRNA) or *mSLC2A9-*specific siRNA and cell lysates run on a Western blot and probed with an antibody specific for either *mSLC2A9a* (mGLUT9a) or *mSLC2A9b* (mGLUT9b). (D) Reduction of urate uptake into MIN6 cells by transfection with siRNA targeted to SLC2A9. MIN6 cells were transfected with either *SLC2A9*-specific siRNA9 or scrambled RNA (scRNA). ^14^C-urate, 120 μM, uptake into transfected or untransfected cells was measured for 6 min at 37 °C. *p < 0.05.

In order to verify the findings in kidney cells, we used a mouse insulinoma cell line (MIN6 cells) endogenously expressing detectable levels of both murine SLC2A9a and SLC2A9b protein. We verified the expression of both mouse SLC2A9a and SLC2A9b protein by using antibodies specifically raised against the N termini of each murine isoform (Figure 5A). We have previously reported SLC2A9 expression in MIN6 cells and islets from both human and mouse [11]. The specificity of these antibodies has been previously reported [11,22]. Mouse SLC2A9a and SLC2A9b in MIN6 cells appear as a doublet with the isoform-specific antibodies as previously shown in both cell lines as well as mouse and ovine tissues [22–24]. The molecular weights are consistent with that published previously for these isoforms in mouse tissue [11].

MIN6 cells take up radiolabelled urate at a rate of 6–7 pmoles/min/mg protein (Figure 5B). In order to test the specificity of SLC2A9a and SLC2A9b for urate, non-radiolabelled urate was added to the uptake assay at a final concentration of 1 mM. The cold urate significantly inhibited radiolabelled uptake by approximately 50% (Figure 5B). These findings suggest that the SLC2A9 isoforms are specific for urate.

### Urate Uptake Is Inhibited by siRNA against SLC2A9 but Not Scrambled RNA

As a final check of the specificity of the SLC2A9 transporter for urate, endogenous expression of mouse SLC2A9a and SLC2A9b in MIN6 cells was knocked down by small interfering RNAs (siRNAs) for both isoforms. Western immunoblotting confirmed a significant decrease in SLC2A9a and SLC2A9b expression in MIN6 cells (Figure 5C). As a result, radiolabelled urate uptake was significantly decreased (Figure 5D) by approximately 50%. Expression and uptake were unaffected by the scrambled RNA.

### Association of *SLC2A9* Gene Variants with Serum and Fractional Excretion of Urate

The Olivetti Heart Study has previously shown strong correlations between uric acid, urate tubular handling, and blood pressure level [12]. We genotyped rs7442295, which had demonstrated the strongest support for association with urate in our previous study, and rs13113918, an intragenic SNP in 868 individuals that had been surveyed at two time points (1994–1995 and 2002–2004). After adjusting for age we confirmed association of both variants with serum uric acid level and detected a complementary association of these variants with reduced urate fractional excretion, which fits with common allelic variation reducing urate clearance by the kidney (Table 1). These associations were evident at both 1994–1995 and 2002–2004 time- points, and after adjustment for BMI and after exclusion of subjects on antihypertensive therapy (unpublished data).

**Table 1:**
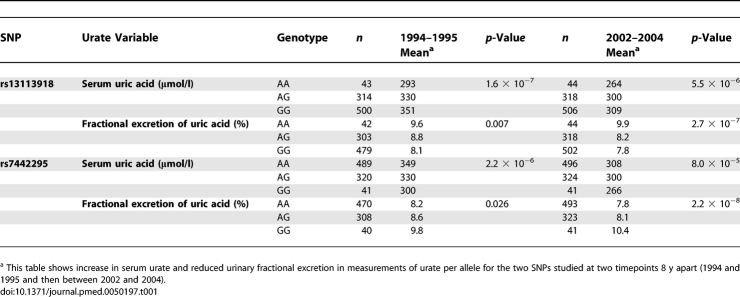
Genetic Association Results for SNPs rs13113918 and rs7442295 in the Olivetti Heart Study in 1994–1995 and 2002–2004 with Serum Uric Acid and Fractional Excretion of Urinary Urate

### Association Studies of the *SLC2A9* Gene with Blood Pressure

For reporting our results here we focus upon the additive model for all cohort analyses, because the association of *SLC2A9* SNPs with serum urate is best described by this model [7]. We performed a quantitative genetic analysis of blood pressure variation with an intragenic SNP rs13113918 in four populations; demographic details for each are summarised in Table 2. In Table 3 we show the analysis of each of these cohorts and the global *p*-value from meta-analysis of systolic and diastolic blood pressure. We found no association with SBP (meta-analysis for SBP showed the effect size of the *SLC2A9* variant to be −0.12 mm Hg, 95% CI −0.68 to 0.44, *p* = 0.66) or DBP (meta-analysis for DBP showed the effect size of the *SLC2A9* variant to be −0.04 mm Hg, 95% CI −0.39 to 0.31, *p* = 0.82) within each population or in meta-analyses under an additive model (Table 3).

**Table 2:**
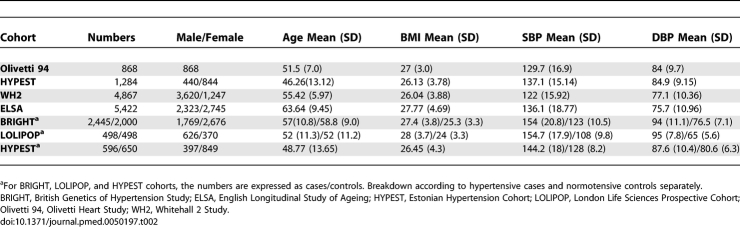
Demographic Characteristics Per Cohort for Genetic Studies

**Table 3:**
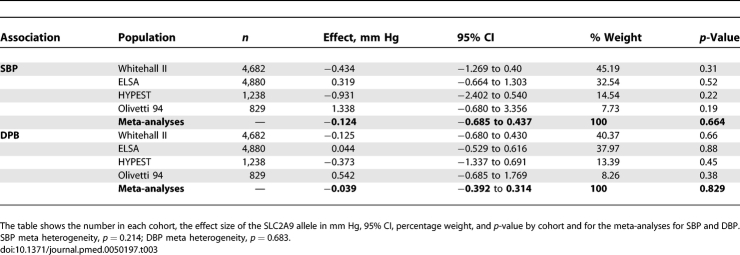
Association of SNPs rs13113918 with SBP and DBP under an Additive Genetic Model of Inheritance

### Association Studies of the *SLC2A9* Gene with Hypertension

We analysed rs13113918 under additive model of inheritance within cases and controls from the BRIGHT study (2,052 hypertensives and 1,637 normotensive controls), the Estonian HYPEST study (596 hypertensives and 650 normotensive controls), the LOLIPOP study (498 hypertensives and 498 normotensive controls), Olivetti 94 (182 hypertensives and 549 normotensive controls), Whitehall II (961 hypertensives and 2,365 normotensive controls) and ELSA (960 hypertensives and 949 normotensive controls) with demographics shown in Table 2. The results of our meta-analysis combining six case–control studies (see Table 4) did not show a significant association between hypertension and the *SLC2A9* variant tested (odds ratio 0.98, 95% CI 0.91 to 1.05, *p* > 0.6).

**Table 4:**
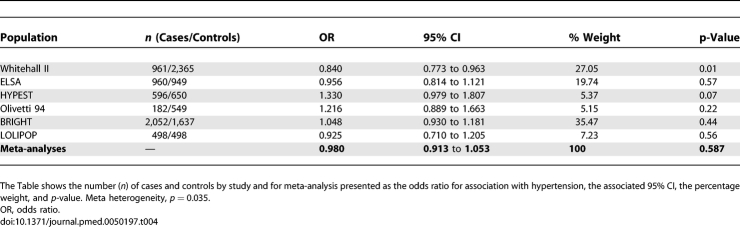
Results of SNP rs13113918 of *SLC2A9* within Individual Hypertensive Case–Control Participants from All Resources and in Meta-analysis Analysed under an Additive Model Testing for Influence on Hypertension

## Discussion

Recent genome-wide association scans identified and replicated association between SNPs at the *SLC2A9* gene locus and serum urate and with gout [6,7,25,26]. In this study we have shown that both human SLC2A9 splice variants, SLC2A9a and SLC2A9b, can mediate urate fluxes at a very high rate and significantly faster than their facilitated transport of either glucose or fructose. The kinetics indicate that the transporter's apparent capacity for substrate, or *K*_m_ value (∼ 1 mM), is above the basal, physiologic plasma concentrations of urate. Moreover, the *V*_max_ value indicates a high-capacity transporter. All of these data suggest that this membrane protein plays an important role in the handling of urate in the proximal nephron, which completely fits with the findings from genome-wide scans of common allelic variation elevating urate by 20 μmol/l per allele [7]. In the context of everyday clinical practice this genetic influence on urate is equivalent to 5%–10% of the normal range of serum urate (180 μmol/l to 420 μmol/l), which is not trivial. These findings are confirmed by complementary functional studies on SLC2A9 in relation to urate handling and gout [25–27].

Current models of urate handling in the proximal convoluted tubule indicate that several types of transporter are involved in the fluxes of urate across both the apical and the basolateral membranes of the epithelial cells. In the apical membrane, these transporters include URAT1, a urate/lactate exchanger that mediates urate movement from urine to epithelium [28]; OATv1, a putative voltage dependent organic anion transporter [29]; MRP4, an ATP driven pump [30]; and UAT, a postulated urate channel [31]. At the other pole of the cells two of the organic anion exchangers, OAT1 and OAT2, present in basolateral membrane, are thought to be able to handle urate, but their physiological role remains to be confirmed [32,33]. Therefore at present there is a well-defined absorptive route across the apical membrane via UAT1 and a secretory route via MRP4, while the means by which urate can either leave the renal epithelium and enter the blood or move in the opposite direction across the basolateral membrane remains to be confirmed.

Recently, it has been proposed that both SLC2A9 proteins are high-affinity glucose/fructose transporters. However, when compared with the principal members of the *SLC2A9* gene family their transport capacity (*V*_max_) is very low [11,34]. We now have evidence that urate is a preferred substrate for both human SLC2A9 variants and for the mouse orthologue. The ability of SLC2A9 to exchange urate with glucose in the absence of competition between these two substrates when present on the same side of the membrane indicates that the protein has separate binding sites. This is not a unique phenomenon in exchange proteins, as the glycerol-6-phosphate transporter exchanges glycerol-6-phosphate for inorganic phosphate [35].

This ability of SLC2A9 to exchange glucose and, to a lesser degree, fructose for urate may be physiologically important. The renal proximal nephron plays a major role in the reabsorption of glucose from the urine using a combination of sodium-coupled hexose transporters, SGLT1 and SGLT2, and members of the SLC2A family, GLUTs 2, 5, and possibly 9 [36–40]. Furthermore, the proximal convoluted tubular epithelium is a major site of gluconeogenesis, converting pyruvate to glucose, which is then released across the basolateral membrane into the blood [41–43]. Our data showing that SLC2A9a can exchange glucose for urate suggest that this protein might play an important role in the secretion of urate from the blood into the urine. Glucose in the urine could exchange for urate in the proximal convoluted tubule epithelial cells across the apical membrane, resulting in the release of urate back into the urine. In addition, glucose in the epithelial cells resulting both from reabsorption and neogenesis could exchange for plasma urate across the basolateral membrane, promoting the accumulation of urate in the cells. Furthermore, such a mechanism could explain the known correlation between the glycosuria seen in diabetes and the reduction in plasma urate levels. The increased glucose in the urine could accelerate the SLC2A9-mediated urate efflux across the apical membrane of the proximal convoluted tubule, but further work is needed to confirm this hypothesis.

Therefore, our findings, in combination with epidemiologic data showing correlation of elevated serum urate with diabetes, metabolic syndrome, obesity, and hyperinsulinaemia, provide a potential mechanism for these associations that warrants further investigation [44–46]. In this context it is of particular interest that recent data from the Health and Nutrition Survey shows correlation between consumption of sugar-based soft drinks with serum urate levels and gout, which might be partly explained by sugar-facilitated uptake of urate by SLC2A9 isoforms [47].

Probenecid and benzbromarone are uricosuric drugs that inhibit renal uptake of urate via URAT 1 on the apical proximal nephron membrane [5]. We found that probenecid had no effect on urate uptake into SLC2A9a-expressing oocytes, whereas benzbromarone showed a dose-dependent inhibition. The significant inhibition of urate transport by 10 and 100 μM benzbromarone implies that there are common features in the binding sites for urate in both URAT1 and SLC2A9a. When in clinical use benzbromarone promoted the loss of urate in the urine; this is believed to be a consequence of an inhibition of uptake across the apical membrane mediated by URAT1 [2]. In contrast, the high *K*_i_ of 27 μM for benzbromarone action on SLC2A9 suggests that at therapeutic doses this drug would have a minimal effect on urate transport mediated by this protein and so does not affect urate secretion.

The Olivetti Heart Study has previously found a strong longitudinal relationship of serum urate levels with blood pressure [48]. The authors also reported a correlation of urate with reduced fractional clearance of lithium as an index of sodium reabsorption in the proximal nephron. This finding could reflect sodium retention and offer a mechanism for previously reported associations of urate and blood pressure [12]. The two SNPs genotyped in the Olivetti cohort validate association of the *SLC2A9* locus with serum urate and demonstrate the association of both SNPs with reduced urinary urate excretion at two time points 8 y apart. This provides additional support for our hypothesis that *SLC2A9* variants reduce urinary urate loss and is further confirmed by recent cross-sectional findings for this gene in gout [25–27].

In view of the additive influence of alleles of *SLC2A9* on serum uric acid and urinary urate excretion, we explored a relationship between *SLC2A9* SNPs with systolic and diastolic blood pressure in 11,629 individuals under an additive model. This analysis showed no significant association between SNP rs13113918 and blood pressure. Furthermore, we did not find any evaluated association of the same *SLC2A9* SNP with hypertension under an additive model in 5,249 hypertensive cases and 6,648 normotensive controls drawn from all the studies. In the context of this large-scale case–control and population-based cohorts we conclude that there is no support for an association with blood pressure using the most biologically plausible genetic model.

### Limitations of This Study

We have not defined precisely the causative variant of *SLC2A9* responsible for elevated serum urate and reduced urinary urate clearance. There are several known SNPs within the gene region that might influence function of the SLC2A9 protein. Such studies will be facilitated by detailed re-sequencing of the *SLC2A9* gene to establish a comprehensive inventory of genetic variation across this locus. In addition to dietary and metabolic influences on uric acid levels there will be other, as-yet unidentified genetic influences on serum urate level that may contribute to epidemiologic correlations with metabolic syndrome, diabetes, gout, and cardiovascular disease.

### Conclusion

In this paper we have translated the genetic association of the *SLC2A9* locus with serum urate derived from genome-wide scanning into a functional confirmation that SLC2A9 splice variants acts as a high-capacity urate transporter that can be facilitated by exchange with hexoses and inhibited by high concentrations of some uricosurics and siRNA technology. These findings offer novel potential pathogenic mechanisms and new drug targets for diseases such as gout.

## Supporting Information

Figure S1 Expression of SLC2A9a or 9b in *Xenopus* Oocyte Plasma Membrane after cRNA Injection Oocytes were injected with 50 nl (1 ng/nl) SLC2A9a or SLC2A9b cRNA or water and incubated at 18 °C for 4 d. The expression of SLC2A9a and SLC2A9b was visualized using a primary antibody to the same C-terminal peptide sequence of both proteins, a fluorescent secondary antibody, and confocal microscopy. All three images show the *x*, *y*, and *z* within the same focal plane.
(A) Water-injected oocyte.
(B) SLC2A9a.
(C) SLC2A9b.
(204 KB PDF)

## Abbreviations

BMI: body mass index
CI: confidence interval
DBP: diastolic blood pressure
HEK: human embryonic kidney
SBP: systolic blood pressure
SEM: standard error of the mean
