# Supplementary material for: SLC2A9 Is a High-Capacity Urate Transporter in Humans
Source: PLoS Med. 2008 Oct 7;5(10):e197. doi: 10.1371/journal.pmed.0050197 (PMC2561076; doi:10.1371/journal.pmed.0050197)
Supplement: Supplementary file 1 [file pmed.0050197.sg001.pdf]

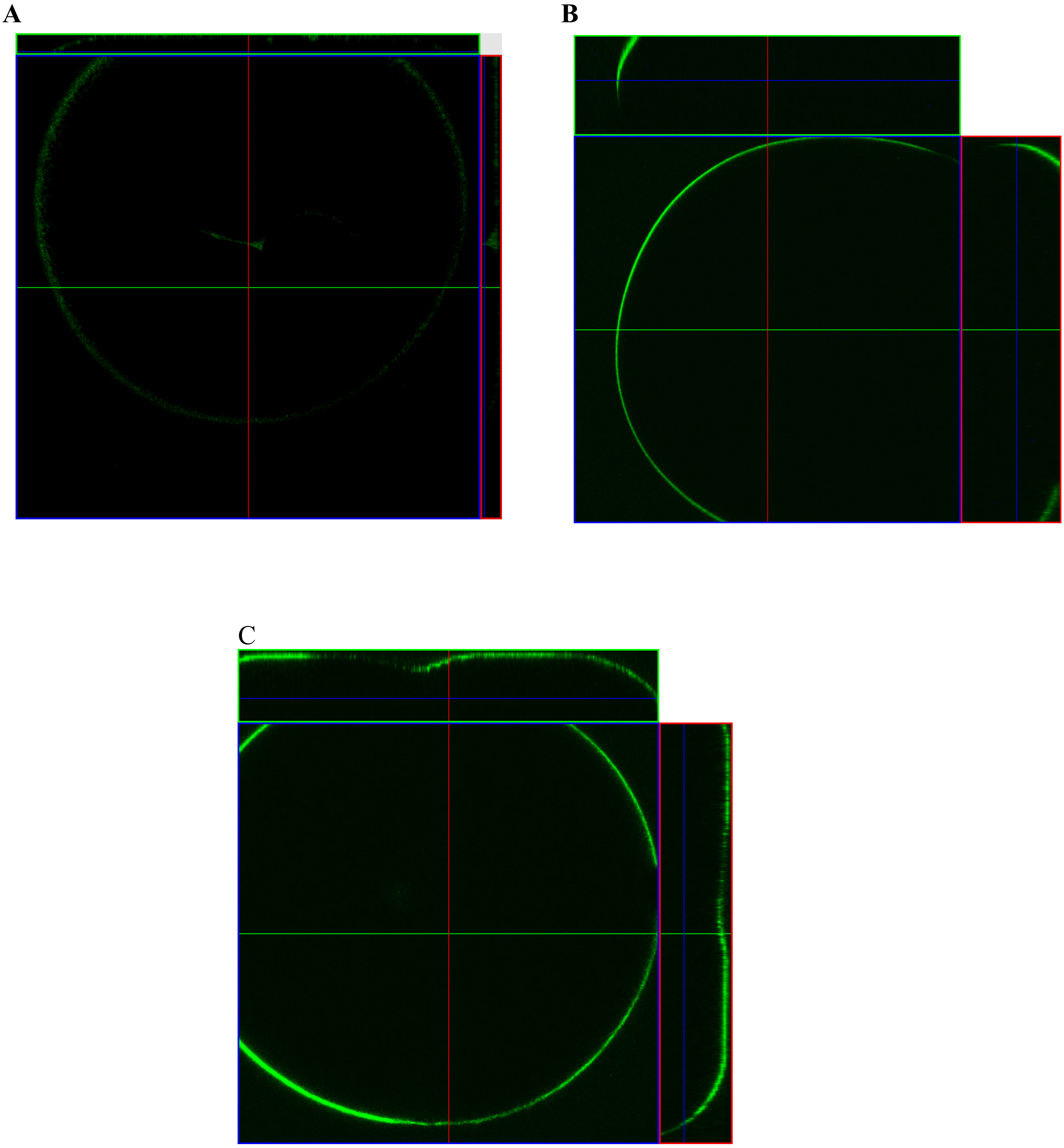
A
B
C
